# Development and Evaluation of Chromosome Segment Substitution Lines Carrying Overlapping Chromosome Segments of the Whole Wild Rice Genome

**DOI:** 10.3389/fpls.2016.01737

**Published:** 2016-11-24

**Authors:** Dewei Yang, Xinfu Ye, Xianghua Zheng, Chaoping Cheng, Ning Ye, Fenghuang Huang

**Affiliations:** Rice Research Institute, Fujian Academy of Agricultural Sciences, Fujian High Quality Rice Research and Development CenterFuzhou, China

**Keywords:** rice (*Oryza sativa L. subsp. indica*), the Zhangpu wild rice (*Oryza rufipogon* Griff.), chromosome segment substitution lines, quantitative trait loci, gene mapping

## Abstract

Common wild rice (*Oryza rufipogon* Griff.) represents an important resource for rice improvement. Genetic populations provide the basis for a wide range of genetic and genomic studies. In particular, chromosome segment substitution lines (CSSLs) are most powerful tools for the detection and precise mapping of quantitative trait loci (QTLs). In this study, 146 CSSLs were produced; they were derived from the crossing and back-crossing of two rice cultivars: Dongnanihui 810 (*Oryza sativa* L.), an *indica* rice cultivar as the recipient, and ZhangPu wild rice, a wild rice cultivar as the donor. First, a physical map of the 146 CSSLs was constructed using 149 molecular markers. Based on this map, the total size of the 147 substituted segments in the population was 1145.65 Mb, or 3.04 times that of the rice genome. To further facilitate gene mapping, heterozygous chromosome segment substitution lines (HCSSLs) were also produced, which were heterozygous in the target regions. Second, a physical map of the 244 HCSSLs was produced using 149 molecular markers. Based on this map, the total length of substituted segments in the HCSSLs was 1683.75 Mb, or 4.47 times the total length of the rice genome. Third, using the 146 CSSLs, two QTLs for plant height, and one major QTL for apiculus coloration were identified. Using the two populations of HCSSLs, the *qPa-6-2* gene was precisely mapped to an 88 kb region. These CSSLs and HCSSLs may, therefore, provide powerful tools for future whole genome large-scale gene discovery in wild rice, providing a foundation enabling the development of new rice varieties. This research will also facilitate fine mapping and cloning of quantitative trait genes, providing for the development of superior rice varieties.

## Introduction

Given the rapid increase in world population, the next century may witness serious global food shortage problems. Consequently, the need to increase grain yield is critical. Meanwhile, rice is one of the most important crops in the world, and in order to meet the growing demand for food driven by population growth and economic development, global rice production must double by 2050 (Arbelaez et al., 2015). This signals the importance of identifying, transferring, and utilizing beneficial allele genes from wild and cultivated rice. Over the past few decades, many different population types have been used to map QTLs, but some factors have impeded the fine mapping or cloning of more QTLs. Researchers have attributed this to several factors, including insufficient population size, unstable statistical thresholds for detecting putative loci, minimal number of molecular markers for analyses, and low heritability of target traits (Yano and Sasaki, 1997). For the most part, however, limited populations prevent fine mapping or cloning.

Early temporary primary mapping populations, such as F_2:3_ and BC_1_ families, have been used for genetic analysis and mapping of target QTLs (Li et al., 1995; Ray et al., 1996; Redona and Mackill, 1996). However, these populations are difficult to maintain, and trials cannot be repeated. Therefore, in order to confirm mapping results, doubled haploid (DH) and recombinant inbred lines (RILs) have been developed. However, neither method is suitable for further analysis, such as fine mapping and cloning of target QTLs (Yano, 2001). For example, separation distortion of RIL populations can occur in groups, actual building construction groups do not necessarily represent theory.

Advanced backcross populations, and NILs have also been developed and used. For example, genetic background noise can be eliminated, and a QTL can be visualized as a single Mendelian factor. Each NIL carries either one or more donor segments in the near-isogenic background of the recurrent parent, which has distinct advantages for QTL identification. Several QTLs have been fine-mapped or cloned on the basis of NILs (Ashikari et al., 2005; Fan et al., 2006; Song et al., 2007; Shomura et al., 2008; Xue et al., 2008; Huang et al., 2009; Fujita et al., 2010; Zhou et al., 2012; Henry et al., 2015). However, since development is trying and time-consuming, many researchers do not perform map-based cloning of QTLs (Xu et al., 2010).

The development of CSSLs, as suggested by Doi et al. (1997) and Kubo et al. (2002), allows QTL detection of complex agronomical traits in plants and may well resolve the issues of precise mapping of QTLs (Li et al., 2015). Specifically, CSSLs can be used for detecting and fine mapping of QTLs as a single Mendelian factor by blocking background genetic noise. So far, several CSSLs in rice have been developed and many QTLs for traits of biological and economic interest have been detected (Kubo et al., 2002; Ebitani et al., 2005; Mei et al., 2006; Takai et al., 2007; Zhu et al., 2009; Xu et al., 2010; Chen et al., 2014; Nagata et al., 2015; Subudhi et al., 2015). These achievements have undoubtedly enhanced the understanding of complex traits and promoted plant genomic studies.

However, rice breeding still faces the problem of yield plateaus and weak resistance, caused by the narrow genetic basis of parental materials (Tanksley and McCouch, 1997; Tian et al., 2006). Sun et al. (2002) compared the genetic diversity between common wild rice and cultivated rice, and the results showed that the number of alleles of cultivated rice was only 60% that of wild rice, indicating that many alleles were lost during the course of domestication from wild to cultivated rice. Therefore, exploitation and utilization of favorable alleles of wild rice previously lost in cultivated rice might overcome the yield plateaus.

The ZhangPu wild rice was found in Zhangpu County in 1982, and it was the only wild rice in Fujian province. However, since it is the easternmost distribution of wild rice in China, it may contain many elite genes, including those for disease resistance, high-yield and resistance to stresses (Li, 2010). This wild rice may contain many genes of use to breeders, such as novel resistance genes to biotic or abiotic stresses, because it was not exposed to selective pressure for all of these traits in its native environment (Furuta et al., 2014). Therefore, systematic and further research on ZhangPu wild rice is needed in order to discover favorable gene resources and enhance our understanding of the molecular basis. Dongnanihui 810 (*Oryza sativa* L.), an *indica* rice cultivar, is an excellent restorer. Ganyou 810, derived from the CMS line Ganxiang A and a restorer line Dongnanihui 810, was certified and released for commercial production in 2014 in Fujian province^1^.

In this study, we described the development of a novel population of CSSLs in rice. The population was derived from the crossing and back-crossing of two rice cultivars: Dongnanihui 810, an *indica* cultivar as the recipient and ZhangPu wild rice, as the donor. Meanwhile a physical map of 146 CSSLs and the 244 HCSSLs was produced on the basis of estimates of lengths and locations using 149 molecular markers. Using the bin map converted from the ultrahigh-quality physical map associated with the purple apiculus of the 146 CSSLs, the *qPa-6-2* gene was precisely mapped to an 88 kb region that contained the *OsC1* gene. This research will facilitate fine mapping and cloning of quantitative trait genes, leading to the development of superior rice varieties. Furthermore, it will be possible to illustrate the genetic mechanisms of complex traits in plant functional genomics.

## Materials and Methods

### Plant Materials

To develop CSSLs and HCSSLs, Dongnanihui 810, a restorer *indica* cultivar, was used as the recipient, and ZhangPu wild rice was used as the donor. The F_1_ plants were generated from Dongnanihui 810 as female and ZhangPu wild rice as male. The F_1_ plants were back-crossed with Dongnanihui 810 to produce the BC_1_F_1_ generation. These BC_1_F_1_ plants were backcrossed to Dongnanihui 810 to produce BC_2_F_1_. In the same way, 156 BC_3_F_1_ individuals were obtained. MAS with a whole-genome survey of 156 BC_3_F_1_ lines, which were selected at random by taking three from each line, identified 213 plants in which most genomic regions were homozygous for Dongnanihui 810 alleles. However, more than four heterozygous substituted segments from ZhangPu wild rice were excluded.

### The Choice of Polymorphism Markers

A lot of 302 SSR markers were selected from dense rice microsatellite maps (McCouch et al., 2002), and 206 InDel markers were developed using Primer Premier 5.0 software according to the publicly available rice genome sequence comparisons between Nipponbare and 9311^2^. Of the 506 markers, 149 (29.4%) displayed better polymorphisms between the two parents.

### PCR Amplification and Marker Detection

Plant DNA was extracted from the frozen leaves of rice plants using the CTAB method (Murray and Thompson, 1980) with minor modifications. The extracted DNA was dissolved in ddH_2_O. DNA amplification was performed by PCR with the following parameters: 5 min at 94°C, 35 cycles of 1 min at 94°C, 1 min at 60°C (for Indel) or 55°C (for SSR), and 50s at 72°C, with a final extension of 10 min at 72°C. For PCR amplification of markers, each 20 μL reaction mixture contained 50 ng DNA, 5 μmol of each primer, 10× PCR buffer [100 mM Tris (pH 8.3), 500 mM KCl, 15 mM MgCl_2_, 2 μg gelatin], 250 μM of each dNTP and 0.5 U of *Taq* polymerase. Amplified PCR products were separated through electrophoresis on a 6% non-denaturing polyacrylamide gel, and bands were revealed using a silver staining procedure.

### Determination of the Length of Substituted Segment in CSSLs

The substituted segment was determined based on its location on the rice microsatellite map (McCouch et al., 2002). The length of substituted segment in CSSLs was estimated based on graphical genotypes (Young and Tanksley, 1989; Zhu et al., 2009). A chromosome segment flanked by two markers of donor genotype (DD) was considered to have a 100% donor genotype, while a chromosome segment flanked by two markers of recipient genotype (RR) was considered to be 0% donor genotype. A chromosome segment flanked by one marker of donor type and one marker of recipient type (DR) was considered as 50% donor genotype. Therefore, the length of DD plus the length of two half DR was considered to be the estimated length of a substituted chromosome segment.

### Identification and Substitution Mapping of QTLs for Purple Apiculus and Plant Height

Dongnanihui 810, ZhangPu wild rice, and 146 CSSLs were grown in a paddy field under natural conditions at the experimental farm of Fujian Academy of Agricultural Sciences (Fuzhou, China), in early of 2015. The field experiment was designed in randomized plots with one plot per genotype. For parents and each CSSL, 60 plants were planted in six rows, and 12 plants in the center of each plot were selected to investigate the characters of purple apiculus. Plant height was the mean value of 6 plants from the middle section of each plot, and QTLs were identified on the basis of significant differences between parents and each CSSL, as determined by *t*-test. All plants were grown according to standard commercial practices, with spacing of 13.3 cm between plants within each row and 26.4 cm between rows. Field management essentially followed normal agricultural practices, and the amounts of N, P_2_O_5_ and K_2_O applied were 127.5 kg/hm^2^, 45.0 kg/hm^2^, and 30.0 kg/hm^2^, respectively.

### Observed Agronomic Characteristics of 146 CSSLs

According to standard commercial practices, 146 CSSLs were grown in a paddy field under natural conditions at the experimental farm of Fujian Academy of Agricultural Sciences (Fuzhou, China). All materials were planted in the same field, the level height of field was consistent, N, P_2_O_5_ and K_2_O were applied in the following amounts: 127.5 kg/hm^2^, 45.0 kg/hm^2^, and 30.0 kg/hm^2^, respectively, and the amount and time of fertilization was uniform. Plant height, panicle length, number of effective panicles, spikelet number per panicle, seed setting rate and 1000-grain weight were all measured at maturity in 2015.

### Physical Map Construction of Target Gene

The physical map of the *qPa-6-2* gene was constructed by bioinformatics analysis using the published sequences of BAC and P1-derived artificial chromosome (PAC) clones of cv. Nipponbare released by the International Rice Genome Sequencing project (IRGSP^3^).

## Results

### Polymorphisms Detected between the Two Parents

**FIGURE 1:**
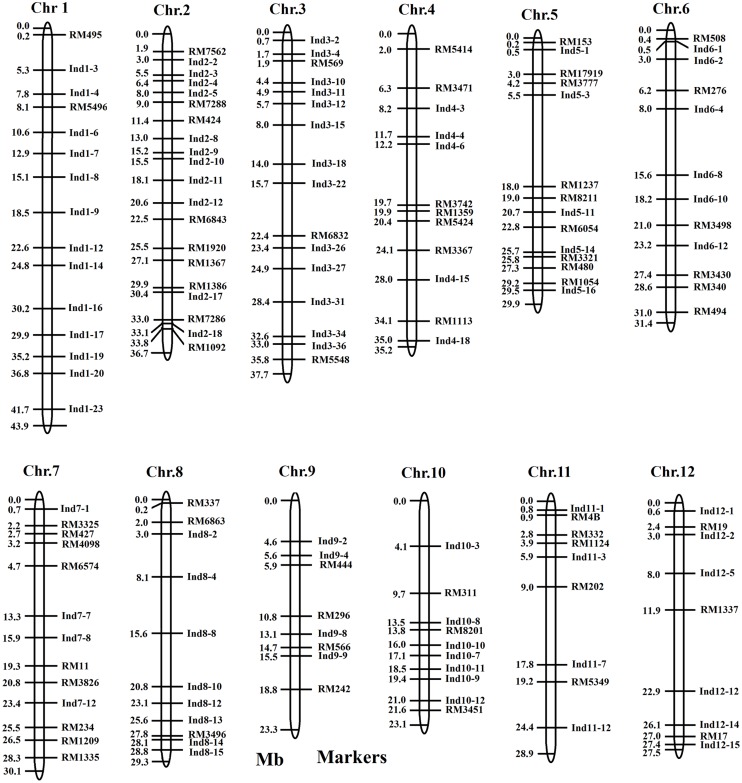
**Locations of the 149 polymorphic markers in the rice physical map**.

Polymorphisms were detected by SSR and InDel markers between the two parents, and were used in this study to survey the polymorphisms between the two parents (**Table 1**). 302 SSR markers were selected from dense rice microsatellite maps (McCouch et al., 2002). A total of 206 InDel markers were developed using Primer Premier 5.0 software according to the publicly available rice genome sequence comparisons between Nipponbare and 9311^4^. Of the 506 markers, 149 (29.4%) displayed better polymorphisms between the two parents (Supplement Table S1). The length of the interval between two polymorphic markers ranged from 0.1Mb to 12.5 Mb, with an average of 2.53 Mb on the rice physical map (**Figure 1**; **Table 1**). The polymorphic markers were utilized further for the further development of CSSLs and HCSSLs.

**Table 1:** Summary of the markers used to develop the CSSLs.

Chromosome	SSR	InDel	Total	Number	Percentage	Density(Mb)
1	31	23	54	15	27.8	2.93
2	35	19	54	20	37.0	1.84
3	27	30	57	16	28.1	2.36
4	24	18	42	12	28.6	2.93
5	24	16	40	14	35.0	2.14
6	26	16	42	12	28.6	2.62
7	23	15	38	13	34.2	2.32
8	27	15	42	11	26.2	2.66
9	19	12	31	8	25.8	2.91
10	24	13	37	10	27.0	2.31
11	22	14	36	9	25.0	3.21
12	20	15	35	9	25.7	3.06
Total	302	206	506	149	29.4	2.53

*SSR, InDel, and Total refer to the number of markers tested on each chromosome while Number refers to the final markers selected on each chromosome.*

### CSSL and HCSSL Development

**FIGURE 2:**
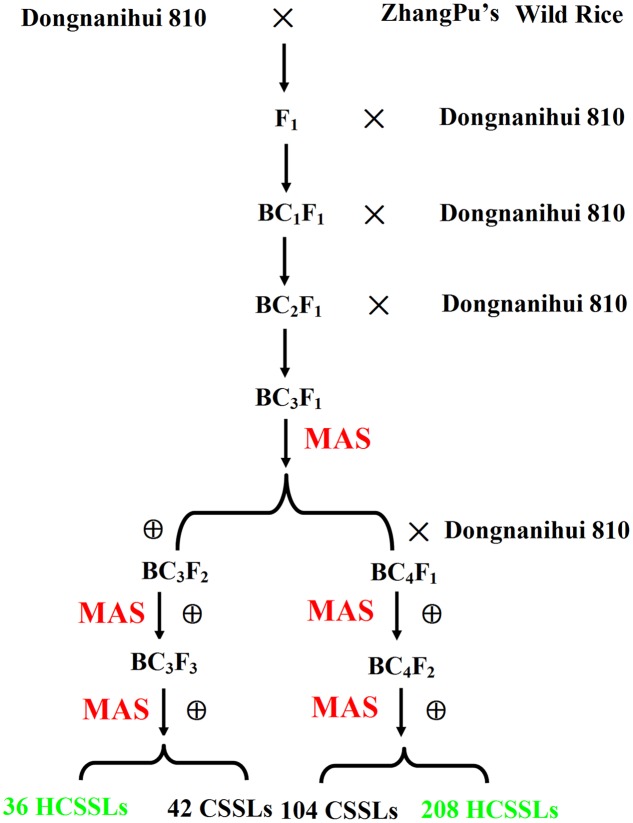
**Flowchart of the development of CSSLs and HCSSLs in the present study**.

The CSSL and HCSSL development procedure was summarized in **Figure 2**. In total, we identified 213 BC_3_F_1_ plants in which the majority of genomic regions were homozygous for Dongnanihui 810 alleles, and 31 BC_3_F_1_ plants that had one substituted segment were self-pollinated to produce thirty-one BC_3_F_2_ lines. Then, 21 plants from each BC_3_F_2_ line were genotyped with the polymorphic markers on the target-substituted segments to select plants that had one homozygous substituted segment from ZhangPu wild rice. Heterozygous substituted segments from ZhangPu wild rice were also selected. As a result, 42 CSSLs were obtained, and 36 HCSSLs were obtained. The remaining 182 BC_3_F_1_ plants, which had two to four substituted segments, were back-crossed with Dongnanihui 810 to produce the BC_4_F_1_ lines, and these plants were self-pollinated to produce BC_4_F_2_ lines. Twenty-one plants from each line were genotyped on the target region to select plants that had one homozygous substituted segment. Forty-five plants from each line were genotyped on the target region to select plants that had two homozygous substituted segments. Ninety-three plants from each line were genotyped on the target region to select plants that had three homozygous substituted segments. At the same time, heterozygous substituted segments from ZhangPu wild rice were selected. As a result, 104 CSSLs were obtained, and 208 HCSSLs were selected. Using these techniques, a total of 146 CSSLs and 244 HCSSLs were selected. Of these CSSLs, eight carried two substituted segments and one carried three substituted segments. Nine HCSSLs, which contained the target region, were planted. Then nine CSSLs were obtained, but only one carried two substituted segments.

### Physical Map of CSSLs and HCSSLs

**FIGURE 3:**
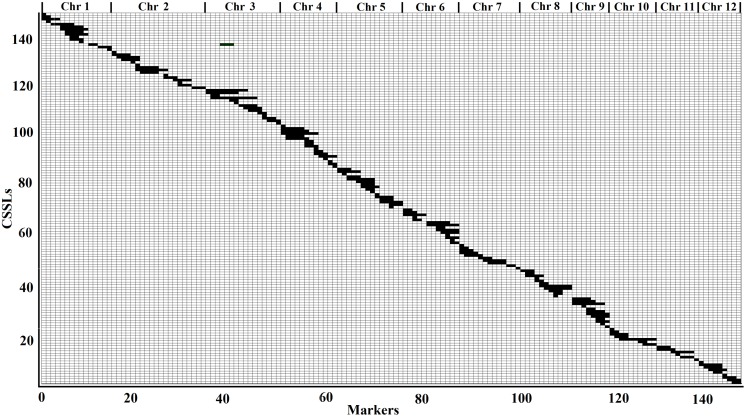
**Physical map of the 146 CSSLs.** Each row represented a CSSL and each column represented a molecular marker locus. The black areas indicate regions that were homozygous genotype for ZhangPu Wild Rice alleles: the white areas indicate regions homozygous for Dongnanihui 810 alleles.

**FIGURE 4:**
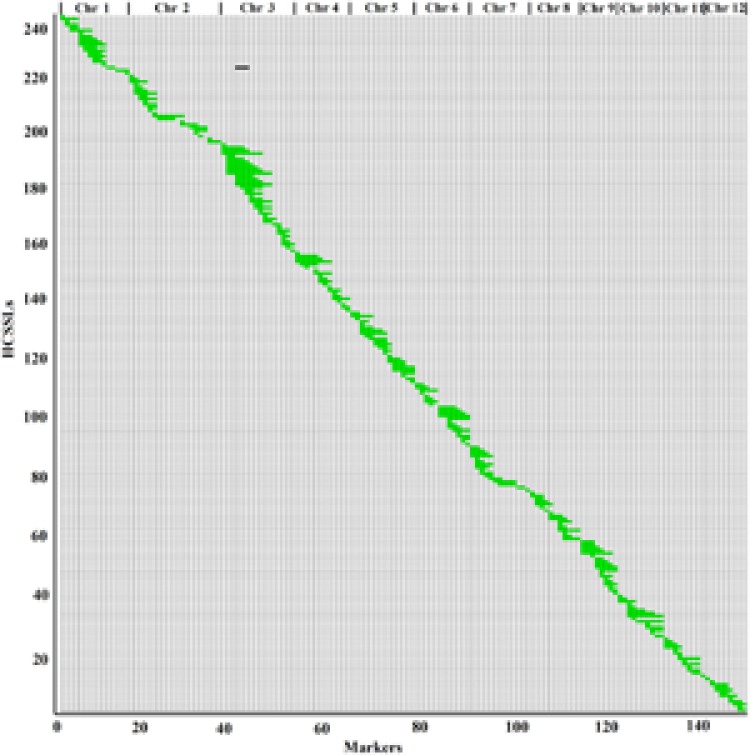
**Physical map of the 244 HCSSLs.** Each row represented a HCSSL and each column represented a molecular marker locus. The black areas indicate regions that were homozygous genotypes for ZhangPu Wild Rice alleles; the white areas indicate regions homozygous for Dongnanihui 810 alleles; the green areas indicate regions that were heterozygous genotypes for Dongnanihui 810 and ZhangPu Wild Rice.

According to the physical locations and genotypes of the 149 molecular markers in the 146 CSSLs, the lengths and locations of the substituted chromosome segments were estimated. Then, using the results of the estimation, a physical map of the 146 CSSLs was constructed (**Figure 3**). In addition, based on 149 molecular markers in the 244 HCSSLs, a physical map, which contained the 244 HCSSLs, was also constructed (**Figure 4**).

### Number, Length and Distribution of Chromosome Substituted Segments in the CSSLs and HCSSLs

**FIGURE 5:**
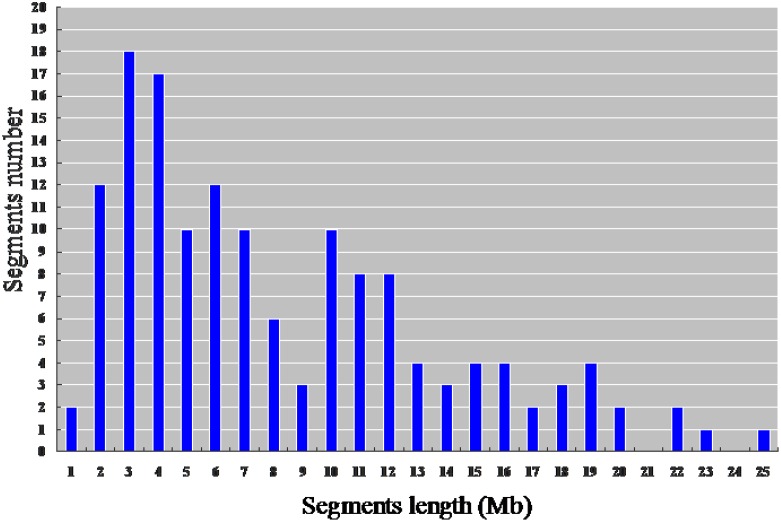
**Distribution of the length of the substituted chromosome segments in the 146 CSSLs based on the physical map constructed with molecular markers**.

From the physical map constructed with molecular markers, the 146 CSSLs carried 147 homozygous substituted segments. Only one CSSL on chromosome 1 carried two substituted segments and the remaining 145 carried only one substituted segment (**Table 2**). In the 146 CSSLs, the length of substituted segments ranged from 550 kb to 24.75 Mb, but averaging 7.79 Mb. The length of 81 substituted segments was shorter than 7.0 Mb and 15 were longer than 16.0 Mb (**Figure 5**). The average number of substitution segments per chromosome was 12.2, but the distribution of the segments was not random among the 12 chromosomes. Different substituted frequencies existed in different chromosomes. For example, 17 existed on chromosome 4, 14 existed on chromosome 1, 4, and 6, while only six existed on chromosome 11 (**Table 2**).

**Table 2:** Segments carried by CSSLs and HCSSLs.

Chromosome	Number of CSSLs		Number of HCSSLs	
	One segment	Two segments	One segment	Two segments
1	14	1	20	1
2	15	0	24	0
3	14	0	38	0
4	17	0	21	0
5	16	0	25	0
6	14	0	22	0
7	10	0	16	0
8	11	0	17	0
9	12	0	19	0
10	7	0	15	0
11	6	0	14	0
12	9	0	12	0
Total	146		244	

**FIGURE 6:**
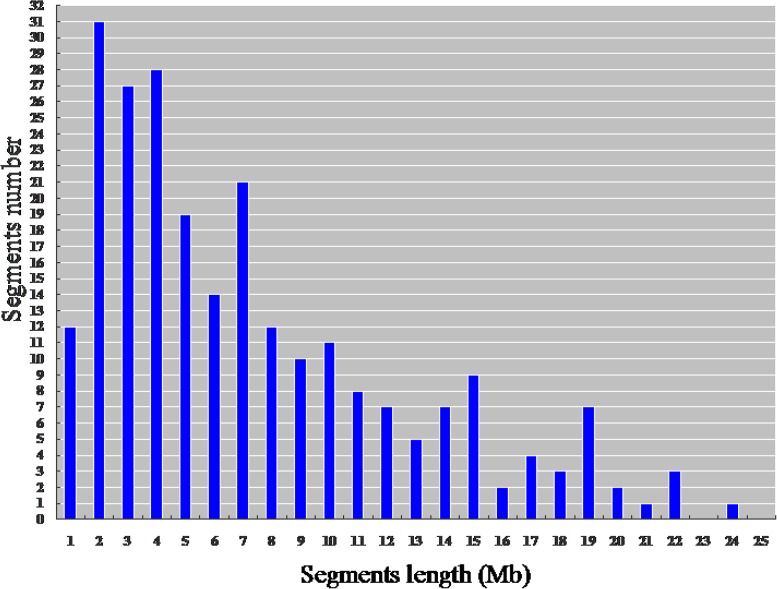
**Distribution of the length of the substituted chromosome segments in the 244 HCSSLs based on the physical map constructed with molecular markers**.

The physical map constructed with molecular markers indicated that the 244 HCSSLs carried 244 heterozygous substituted segments and one homozygous substituted segment. Of these, only one HCSSL carried two substituted segments, which contained one heterozygous and one homozygous segment, while the remaining 243 HCSSLs carried only one heterozygous substituted segment (**Table 2**). The length of heterozygous substituted chromosome segments in the 244 HCSSLs ranged from 300 kb to 23.45 Mb, but averaging 6.90 Mb. Overall, 152 heterozygous segments were shorter than 7.0 Mb, and 21 heterozygous segments were longer than 16.0 Mb (**Figure 6**). The average number of substitution segments per chromosome was 20.3 in the 244 HCSSLs. However, different introgressed frequencies were identified among the 12 chromosomes in that 38 heterozygous segments existed on chromosome 3, while 12 heterozygous segments existed on chromosome 12 (**Table 2**).

### Genome Coverage of Substituted Segments in the CSSLs and HCSSLs

The physical map indicated that the average length of substituted segments per chromosome was 95.47 Mb in the CSSLs, ranging from 44.30 Mb on chromosome 10–144.80 Mb on chromosome 4. The total length of substituted segments in the CSSLs was 1145.65 Mb, which was 3.04 times the total length of the rice genome, and there were different in 12 chromosomes, ranging from 1.65 times on chromosome 11–4.11 times on chromosome 4. All of the chromosomes had 100% coverage in both cases (**Table 3**).

**Table 3:** Chromosome coverage of substituted segments in CSSLs and HCSSLs.

Chromosome	Length (Mb)		Times		Coverage length (Mb)		Coverage rate (%)		Chromosome length (Mb)
	CSSLs	HCSSLs	CSSLs	HCSSLs	CSSLs	HCSSLs	CSSLs %	HCSSLs %	CSSLs
1	136.1	179.90	3.10	4.10	43.90	43.90	100	100	43.90
2	80.40	103.10	2.19	2.81	36.70	36.70	100	100	36.70
3	140.50	340.10	3.73	9.02	37.70	37.70	100	100%	37.70
4	144.80	153.40	4.11	4.36	35.20	35.20	100	100	35.20
5	112.10	160.60	3.75	5.37	29.90	29.90	100	100	29.90
6	101.35	149.70	3.23	4.77	31.40	31.40	100	100	31.40
7	62.20	86.30	2.07	2.87	30.10	30.10	100	100	30.10
8	96.40	114.60	3.29	3.91	29.30	29.30	100	100%	29.30
9	91.20	125.05	3.91	5.37	23.30	23.30	100	100%	23.30
10	44.30	74.35	1.92	3.22	23.10	23.10	100	100%	23.10
11	47.65	86.90	1.65	3.01	28.90	28.90	100	100%	28.90
12	88.65	109.15	3.22	3.97	27.50	27.50	100	100%	27.50
Genome	1145.65	1683.75	3.04	4.47	377.00	377.00	100	100%	377.00

*Length, total length of all substituted segments on a chromosome; Coverage length, length of cover on a chromosome; Times refers to Length/Chromosome length.*

The physical map showed that the average length of substituted segments per chromosome was 140.31 Mb in the HCSSLs, ranging from 74.35 Mb on chromosome 10–340.10 Mb on chromosome 3. The total length of substituted segments in the HCSSLs was 1683.75 Mb, which was 4.47 times the total length of the rice genome, and different frequencies in rice chromosome, ranging from 2.81 times on chromosome 2 to 9.02 times on chromosome 3. The average coverage of substituted segments per chromosome was also 100% (**Table 3**).

### Substitution Mapping of QTLs for Plant Height in the CSSLs

In order to evaluate the potential advantages of the CSSLs for QTL detection, phenotypic variations of plant height were observed in 146 CSSLs.

**FIGURE 7:**
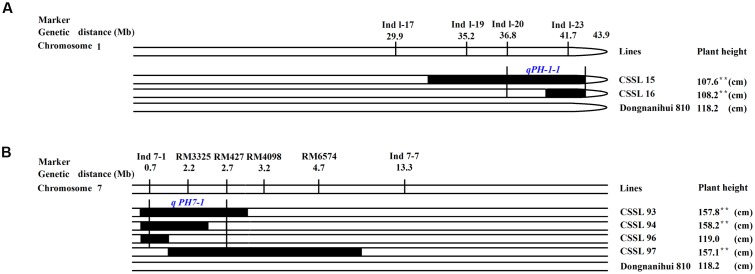
**Substitution mapping of plant height. (A)** Substitution mapping of the *qPH-1-1* gene on rice chromosome 1; **(B)** Substitution mapping of the *qPH-7-1* gene on rice chromosome 7. The substituted segments from ZhangPu wild rice were denoted by dark bars. The substituted segments from ZhangPu wild rice were denoted by black bars with the assumption that a segment flanked by one marker of donor type and one marker of recipient type was 50% donor genotype. Note: ^∗∗^Shows significant at 0.01 level. For the purposes of mapping, however, the full region between one marker of donor type and one marker of recipient type was used as the boundary on each end. The vertical bars through the CSSLs designate the region to which the gene was mapped.

The two parents showed highly significant differences in plant height. Using six CSSLs, two QTLs were identified, in which *qPH-1-1* was mapped in the marker intervals between Ind 1–20 and Ind 1–23 (7.1 Mb) on rice chromosome 1 (**Figure 7A**), while *qPH-7-1* was mapped between Ind7-1 and RM427, which spanned 2.0 Mb in genetic distance on rice chromosome 7 (**Figure 7B**).

### Substitution Mapping of QTLs for Purple Apiculus in the CSSLs

**FIGURE 8:**
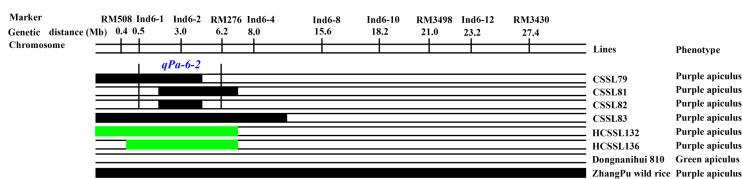
**Substitution mapping of *Pa-6-2* for purple apiculus on rice chromosome 6.** The substituted segments from ZhangPu wild rice were denoted by dark bars; the green areas indicate regions that were heterozygous genotypes for Dongnanihui 810 and ZhangPu Wild Rice; the substituted segments from ZhangPu wild rice were denoted by black bars with the assumption that a segment flanked by one marker of donor type and one marker of recipient type was 50% donor genotype. For the purposes of mapping, however, the full region between one marker of donor type and one marker of recipient type was used as the boundary on each end. The vertical bars through the CSSLs designate the region to which the gene was mapped.

For purple apiculus, ZhangPu wild rice displayed purple apiculus, while the *indica* variety, Dongnanihui 810, displayed green apiculus. Using four CSSLs, one major QTL for purple apiculus was identified, in which *qPa-6-2* was located between Ind6-1 and RM276, which comprised 5.7 Mb in physical distance on rice chromosome 6 (**Figure 8**).

### Genetic Analysis of the *qPa-6-2* Gene for Purple Apiculus

The two segregating populations, HCSSL132 and HCSSL136, which were heterozygous in the target region and showed purple apiculus (**Table 4**), were grown in a paddy field under natural conditions at the experimental farm of Fujian Academy of Agricultural Sciences (Fuzhou, China), in late 2015. Segregating plants were therefore recorded as either purple or green apiculus. The segregation of purple to green apiculus based on 4932 F_2_ plants fitted a ratio of 3:1(χ^2^ = 0.330 ∼ 0.688, *P* > 0.05) (**Table 4**), indicating that the gene for purple was dominant over green color and was controlled by a major gene.

**Table 4:** Plants segregating for purple apiculus.

Serial number	Phenotype	F_2_ population			*χ*^2^(3:1)	P
		Green apiculus	Purple apiculus	Total plants		
HCSSL132	Purple apiculus	603	1839	2442	0.120^a^	<1.0
HCSSL136	Purple apiculus	638	1852	2490	0.310^a^	0.5–0.75

*^a^Denotes the segregation ratio of normal plants to mutant plants complied with 3:1 at 0.05 significant probability level.*

### Fine Mapping of the *qPa-6-2* Gene

**FIGURE 9:**
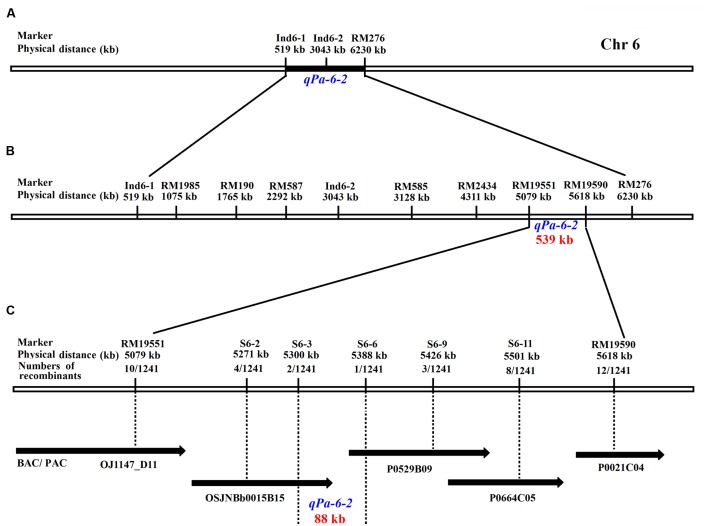
**Genetic and physical maps of the *qPa-6-2* gene.** **(A)** Primary mapping of the *qPa-6-2* gene. The gene was mapped to the region between markers Ind6-1 and RM276; **(B)** Further mapping of the *qPa-6-2* gene. The gene was mapped to the region between markers RM19551 and RM19590; **(C)** Fine mapping of the *qPa-6-2* gene. The *qPa-6-2* gene was finally defined in an 88 kb region, and the recombinants number between markers and target gene was indicated under the physical distance.

To map the gene to a smaller region, 1241 recessive individuals were identified from the two HCSSL populations (**Table 4**). A higher precision map was constructed using published markers^5^ in the region between Ind6-1 and RM276 (**Figure 9A**). All recombinants were genotyped using seven polymorphic markers. The results showed that the *qPa-6-2* gene was mapped between molecular markers RM19551 and RM19590 on chromosome 6 and that the physical distance between the two markers was 539 kb with a physical distance (**Figure 9B**; **Table 5**).

**Table 5:** Simple sequence repeats and Indel molecular marker developed for *qPa-6-2* gene mapping.

Marker	Sequence of forward primer	Sequence of reverse primer	Locations
RM1985	TCATACCCATTTAAATTGAG	GTTTGAAGCAAGTACAAAAG	OSJNBa0062J13
RM190	CTTTGTCTATCTCAAGACAC	TTGCAGATGTTCTTCCTGATG	OSJNBa0007O20
RM587	ACGCGAACAAATTAACAGCC	CTTTGCTACCAGTAGATCCAGC	OSJNBa0007O20
RM585	CAGTCTTGCTCCGTTTGTTG	CTGTGACTGACTTGGTCATAGG	P0681F10
RM2434	CATATCACCCAGAATTCTAA	AAGAGATTTAAGTTGCACTC	P0568D10
RM19551	CCCACCAGCTGCTACTTTGTGC	CGCCAGGAAGTCGAGGATAGG	OJ1147_D11
S6-2	CATCTGATCTCGCATGCACTTGG	GTCTCTCTGCCGCTGGATCG	OSJNBb0015B15
S6-3	TTGTGGTTGTAGTGTGCTTGTGC	CGGAACGAGAGGACAATGTACG	OSJNBb0015B15
S6-6	GGAGGTTCGAGTGCCACTACTGC	AAAGCACCACCACCACCACTCC	P0529B09
S6-9	TCCTTCAAGAGTGCAAAACC	GCATTGTCATGTCGAAGCC	P0529B09
S6-11	ACTTCGACGTCAGGTTCGACACG	CCGCCTCAAGGAAGAGGTAATGC	P0664C05
RM19590	CAATCCCGAGCCTAAACCAAACC	GCTGGATCTCCTCGGACACG	P0021C04

To fine map the *qPa-6-2* gene, five polymorphic InDels were selected from 12 new InDels (**Table 5**). The InDel markers were designed from the publicly available rice genome sequences, and the likelihood of detecting polymorphism between ZhangPu wild rice and Dongnanihui 810 was predicted by comparing sequences from *Nipponbare*^6^ and *Indica* cultivar 93-11^7^. Firstly, the BAC clone’s sequences of *japonica* and *indica* were aligned, then primers were designed using Primer premier 5.0 based on the polymorphism region between the two rice subspecies and the polymorphic markers were used for gene mapping. Recombinant screening with five markers (S6-2, S6-3, S6-6, S6-9 and S6-11), which were more internal to the *qPa-6-2* locus, detected four, two, one, three and eight recombinants, respectively. Thus, the *qPa-6-2* gene was precisely mapping in an 88 kb region by S6-3 and S6-6 (**Figure 9C**).

## Discussion

### Excellent Resources in Wild Rice

*Oryza Rufipogon* Griff., a wild rice, is the progenitor of the cultivated rice *O. sativa* L. As such, it is well recognized as a natural gene bank that conserves many specific genes. During the course of domestication from common wild rice to cultivated rice, profound changes in morphology and molecular genetic profile occurred via natural and artificial selection. Over the years, polymorphisms have been identified between wild and cultivated rice, including some that affect yield, disease resistance, or insect resistance (Tian et al., 2006; Jena, 2014).

Major resistance genes have been transferred to cultivated rice from *O. nivara* and *O. longistaminata* (Khush, 1977; Khush et al., 1990). A new gene, *Xa-23*, which showed resistance to bacterial blight was discovered and cloned from common wild rice of Guangxi (Wang et al., 2014, 2015). The new resistance gene for *Magnaporthe oryzae*, *Pi9*, was identified from wild rice (Zhuang et al., 1998; Jiang et al., 2012). The major gene for *Magnaporthe oryzae, Pi40*, was discovered from the wild rice *O. australiensis* (Jeung et al., 2007). The new genes, *Bph14* and *Bph15*, which showed resistance to the brown planthopper, were identified from the wild rice (*Oryza officinalis* Wall) (Yang et al., 2004; Du et al., 2009). Meanwhile, Li et al. (2002) identified two high-yielding QTLs from Dongxiang common wild rice of China. Tan et al. (2008) cloned the *PROG1* gene from common wild rice, and *prog1* variants identified in *O. sativa* disrupt *prog1* function and inactivate *prog1* expression, leading to erect growth, greater grain number and higher grain yield in cultivated rice. At the same time, the flowering time QTL *dth3*, was detected from wild rice species (Bian et al., 2011), and the seed dormancy QTL was also isolated in the genetic populations derived from wild rice species (Gu et al., 2004).

### CSSLs as a Platform for QTL Mapping and Cloning

Chromosome segment substitution lines were a series of NILs in which the substituted segments of containing the entire information of the donor, and each CSSL carried one or more donor chromosome segments. Since the main characteristic of CSSLs is that the substituted segment of each CSSL was homozygous and stable, they were useful for genetic studies and fine mapping of QTLs for genome-wide target traits. In the present study, we developed 146 CSSLs in the genetic background of rice restorer Dongnanihui 810. The 146 CSSLs had different agronomic traits, including, for example, long grain, plant height, panicle length, effective panicle, and long flag leaf. Using these lines, we mapped two QTLs for plant height. The *qPH-1-1* mapped in this study was probably an allele of *sd1* (Sasaki et al., 2002; Ye et al., 2015), while *qPH-7-1* might be a novel QTL. The *qPH-1-1* mapped between the markers Ind1-20 and Ind1-23 in this study, which contained the *sdl* locus, a gene known to play a significant role in height in rice. These results demonstrated that the uniformity of the genetic background of the lines facilitated the identification of a number of QTLs by direct comparison between each line and the recipient parent. For further fine mapping and positional cloning of interesting QTLs, secondary F_2_ populations could be derived from a further back-cross between the target CSSL and the recurrent parent (Frary et al., 2000; Yano et al., 2000). Therefore, CSSLs simplified the process of data analysis and increased the accuracy of the results. In this study, using the 146 CSSLs, one major QTL for purple apiculus was identified. Here, the *qPa-6-2* was located between two markers Ind6-1 and RM276 on rice chromosome 6. More importantly, the *qPa-6-2* gene was precisely mapped to an 88 kb region, which contained the rice *OsC1* gene (Saitoh et al., 2004). The occurrence of varying degrees of apiculus coloration (also shows purple apiculus) due to anthocyanin pigmentation, which was reported to be caused by a series of alleles at the C locus, and the C gene appears to be the rice homolog (*OsC1*) (Saitoh et al., 2004). In our study, we identified the qPa-6-2 for purple apiculus, which was also located in the same region. Meanwhile, the *Pa-6* gene was also mapped to the same region with a genetic distance of 41.7 kb (Liu et al., 2012). It is well known that male sterile lines play very important in hybrid rice. Meanwhile, purple apiculus is helpful to increase outcrossing rate of male sterile lines in rice. Therefore, fine mapping and positional cloning of purple apiculus are of great significance in hybrid rice breeding.

### The CSSLs as a Platform for Rice Molecular Breeding

Plant breeding, which aimed to improve the genetic basis of new varieties of crops with increased productivity and quality, combines art with science (Xi et al., 2006). In a general way, traditional breeding was predominantly based on phenotypic assays. Facilitated by the recent developments in genome sequencing, molecular markers and bioinformatics, plant breeding systems entered an era of molecular breeding, shortening the breeding period, improving efficiency, and overcoming the traditional shortcoming of low accuracy with broad applications. Thus, it became the standard in plant breeding programs to combine phenotype and genotype (Xu et al., 2010).

However, this approach only targeted the QTLs with very few traits for genetic improvement. On the other hand, CSSLs, which selected at the level of the whole genome with multi-trait breeding objectives, have expanded targets and thus become important in improving the properties of plants (Xu et al., 2010). Therefore, CSSLs for rice breeding were considered as a key tool in the ongoing technological innovation of plant breeding (Xu et al., 2010).

The *indica* cultivar Dongnanihui 810, used as the recipient in this study, has been planted on a large scale as an excellent restorer. Ganyou 810, derived from the CMS line Ganxiang A and a restorer line Dongnanihui 810, was certified and released for commercial production in 2014 in Fujian province. The ZhangPu wild rice, used as the donor in the present study, was the only wild rice in Fujian province. Interestingly, eight elite CSSLs in this wide population had a genetic background similar to that of Dongnanihui 810, but their agronomic traits were better than those of Dongnanihui 810 (**Table 6**). The effective panicle, each panicle contains at least five seeds (including 5) in per plant, was one of the important indicators of yield traits. For example, CSSL15 had lower plant height and more effective panicle than Dongnanihui 810. CSSL42, CSSL102 and CSSL123 had higher seed setting rate, and CSSL53 had more effective panicle. CSSL77 and CSSL78 had longer panicle and more spikelets, while CSSL102 and CSSL138 both had higher 1000-grain weight. None of these eight CSSLs had poor agronomic traits. Therefore, they could be used to create new varieties with direct marketing applications and, as a parent, create new hybrids. Furthermore, favorable alleles could be combined through MAS to improve the production of superior rice varieties.

**Table 6:** Comparison of main agronomic traits among eight CSSLs and Dongnanhui 810.

Name	Plant height (cm)	Panicle length (cm)	Number of effective panicles	Spikelets per panicle	Seed setting rate (%)	1000-grain weight (g)
Dongnanhui 810	118.2	24.8	8.0	127.2	92.79	30.25
CSSL15	107.6^∗∗^	22.1^∗^	13.2^∗∗^	121.4^∗^	93.47	29.67
CSSL42	115.2	24.2	8.6	124.2	98.25^∗∗^	29.17
CSSL53	119.1	25.2	10.6^∗∗^	126.2	92.04	30.55
CSSL77	116.8	30.9^∗∗^	8.2	142.2^∗∗^	92.46	30.45
CSSL78	117.2	31.2^∗∗^	7.9	146.4^∗∗^	91.94	29.95
CSSL102	119.2	24.3	7.8	125.7	94.74^∗^	34.17^∗∗^
CSSL123	116.8	25.2	8.2	126.6	97.48^∗∗^	30.15
CSSL138	119.0	25.2	850	126.7	92.99	32.45^∗^

*^∗^Shows significant at 0.05 level; ^∗∗^Shows significant at 0.01 level; The seed setting rate was filled grains per panicle /total grains per panicle × 100%.*

### Why Should We Develop the HCSSLs in This Paper?

HCSSLs showed technological innovation in rice genetic research. Up to now, after developing CSSLs in rice, many HCSSLs, which carried one or more donor heterozygous chromosome segments, were discarded. However, for further fine mapping and positional cloning of interesting QTLs, secondary F_2_ populations were reconstructed, which should be derived from a further back-cross between the target CSSL and the recurrent parent. Obviously, this not only increased experimental cost, but also reduced efficiency. In the present study, 244 HCSSLs were produced on the basis of estimates of lengths and locations, and the total length of substituted segments among HCSSLs was 1683.75 Mb, or 4.47 times the total length of the rice genome.

It was convenient to perform gene mapping and analysis of genetic characteristics for agronomic traits using the HCSSLs. In this paper, using the 146 CSSLs, the major *qPa-6-2* for purple apiculus was identified. To confirm this, further analysis showed that both HCSSL132 and HCSSL136 were heterozygous in the target region and showed purple apiculus. Meanwhile, HCSSL132 and HCSSL136 were planted. Further observation of these two populations showed that the gene for purple apiculus was dominant over green and was controlled by a major gene. Importantly, without reconstructing secondary F_2_ populations, the *qPa-6-2* was precisely mapping in an 88 kb region using the two HCSSLs populations.

It was easy to improve the development of CSSLs carrying overlapping chromosome segments of the whole rice genome using HCSSLs. From a genetics perspective, the number of heterozygous plants, as a separate population, was nearly twice that of homozygous plants. Regrettably, only homozygous substituted plants were selected, while many heterozygous substituted plants were discarded. As a result, CSSLs could barely cover the whole genome of donor parents. However, incomplete coverage of a donor genome in the CSSL population might, in turn miss some QTLs responsible for useful traits (Subudhi et al., 2015). In this paper, with the help of HCSSLs, CSSLs carrying one substituted segment could be obtained. At the early stage of constructing CSSLs, although 146 CSSLs were obtained in this paper, eight of these CSSLs carried two substituted segments and one carried three substituted segments. Using nine HCSSLs containing the target region, nine CSSLs were obtained, which only one CSSL carried two substituted segments, the rest of the eight carried one substituted segment.

## Author Contribution

DY drafted the manuscript. DY and FH participated in the development of chromosome segment substitution lines. DY, XZ, CC, and NY contributed to data analysis. DY and XY participated in the design of the study and the interpretation of the results and wrote and edited the manuscript.

## Conflict of Interest Statement

The authors declare that the research was conducted in the absence of any commercial or financial relationships that could be construed as a potential conflict of interest.

## Footnotes

1 http://www.ricedata.cn/variety/varis/614685.htm

2 http://rgp.dna.affrc.go.jp

3 http://rgp.dna.affrc.go.jp/IRGSP/index.html

4 http://rgp.dna.affrc.go.jp

5 http://archive.gramene.org/markers/

6 http://rgp.dna.affrc.go.jp/

7 http://rice.genomics.org.cn/

## Abbreviations

Ls: chromosome segment substitution lines
HCSSLs: heterozygous chromosome segment substitution lines
INDEL: insertion/deletion
MAB: marker-assisted breeding
MAS: marker-assisted selection
NILs: near-isogenic lines
QTLs: quantitative trait loci
SSR: simple sequence repeats
