# Supplementary material for: Development and Evaluation of Chromosome Segment Substitution Lines Carrying Overlapping Chromosome Segments of the Whole Wild Rice Genome
Source: Front Plant Sci. 2016 Nov 24;7:1737. doi: 10.3389/fpls.2016.01737 (PMC5121215; doi:10.3389/fpls.2016.01737)
Supplement: Supplementary file 1 [file Table_1.DOC]

**Supplement material:**

**149 markers displayed better polymorphisms between the two parents**

| Marker | Sequence of forward primer | Sequence of reverse primer | Chromosome |
| --- | --- | --- | --- |
| RM495 | AATCCAAGGTGCAGAGATGG | CAACGATGACGAACACAACC | 1 |
| Ind1-3 | GGGAAATTTGGGAGGAAGAC | CGAGCAAGCTACCCGAATTA | 1 |
| Ind1-4 | CAGCATACAGTACGCATCATCA | GCTCGTATCTCGATGAGTCCA | 1 |
| RM5496 | TGCCTACTCAGCAACTAACAC | ACTTTGCAGTTTGCACATC | 1 |
| Ind1-6 | TGATGGTACAGCCACTTGGT | CGTACGATTGTCCTCGTCCT | 1 |
| Ind1-7 | ATTCCTGGTTCTACATTACTTA | CGCCTCACTAGAATATCGGA | 1 |
| Ind1-8 | TGGCCCAATAGCCCATTTAT | CGAGAGCCCGAGAGAGAGA | 1 |
| Ind1-9 | TTGGAACAGGGAAGAAGC | AGGACATAGTTGTAATGGGTAG | 1 |
| Ind1-12 | GTTCAGGCAATTCCATCGTT | CTAATTCGCGAAACGAATCT | 1 |
| Ind1-14 | AAGGGGCCCTAATTTATCTA | TGTTTACTTTGTTCTTGGACTG | 1 |
| Ind1-16 | CGGTTGGTCATGAACTTGC | CAGCTACAAGCCCACCGTAT | 1 |
| Ind1-17 | ATAGTTCGCCATCGTCAT | ACACGCCATAGCAAGGAA | 1 |
| Ind1-19 | CAACCCCTCCAAATACCTGA | ACCGTGTTCATGCCTTTCAC | 1 |
| Ind1-20 | AATAGAATTACTGATGAAACCTTA | GCCCGTTACCGCTTATGT | 1 |
| Ind1-23 | TTCATATCCGCAGGCAATTT | GCCTTTCTGTTCATGGCAGT | 1 |
| RM7562 | AGACATGCCAATGTGATGGC | TCGGTAGTATGGGGCTTGTC | 2 |
| Ind2-2 | CACATGCTCTGGACACCAAC | GGAGCAAATAAGCCAACCAA | 2 |
| Ind2-3 | ATAGGGTGGGTGTGCTGAAC | GCACAAAACTGCAGGTCTCC | 2 |
| Ind2-4 | CCCAGTCTGCTGCCATCT | GAATGTATTTCAGTTCCAGTAAG | 2 |
| Ind2-5 | AGTGTCCCAAGCGAGAAAAC | ATGCACGAGTGAGTGTGAGC | 2 |
| RM7288 | TTTCTCAACTGAAACAACAT | AGTTTAAGAGCGTTTCTAGG | 2 |
| RM424 | TTTGTGGCTCACCAGTTGAG | TGGCGCATTCATGTCATC | 2 |
| Ind2-8 | TACCTCGGCTCGGGTCAAT | CGACCAAGCGAGAAGGTACT | 2 |
| Ind2-9 | GCAGCAAAGTGCGGAGTA | CAGGTGAATTGCCAATTT | 2 |
| Ind2-10 | GAACCAGTCCGCTCTCTGAC | TACGCGTCGTGTATCGTAGC | 2 |
| Ind2-11 | CCATCAGCATCAGCAATAGG | GAATATGTTGTGGAGACCAATATATGA | 2 |
| Ind2-12 | TTTTTATCTCGAGGTACGGTAGTAG | TGAGAATTAGTGTTCATTTGTAGATTC | 2 |
| RM6843 | gacaaattcagctgttgacc | ataaaccacaatgagcaagc | 2 |
| RM1920 | CAAACACAGTGTTGACAGAA | GCTATTGACTTATCCGTTCA | 2 |
| RM1367 | GTGTGTACGTAGGATCGGAG | TGCTACTCCTAGCTGCTACC | 2 |
| RM1386 | ATACATGCATACTCCGATCC | TCGAGTTGTGAAAGATAGGG | 2 |
| Ind2-17 | CCTGAAGGAAATGATAGCAATAG | GTTTTGTATGCTCTTCACTTGTC | 2 |
| RM7286 | CAGAACAATTCGACCGCTTC | GGCTTGAGAGCGTTTGTAGG | 2 |
| Ind2-18 | AGTGAAATTTGAGCCCAACG | TAAAAGCAAAGGCCGAAAAA | 2 |
| RM1092 | ACCCACCACCCTTTGACC | ATTTTGGTCTACGTGACGGC | 2 |
| Ind3-2 | TACTTTAATTTTGCAGCTC | TTTTACCCCACTCCATCT | 3 |
| Ind3-4 | GCTTACCACACCTCTCCTCCT | TCCATATGCTTCCTTCTTCCA | 3 |
| RM569 | GACATTCTCGCTTGCTCCTC | TGTCCCCTCTAAAACCCTCC | 3 |
| Ind3-10 | CAGGCCGGATCTAGTTGAAA | CAAAGTGAACAGGCTCGAGAT | 3 |
| Ind3-11 | ACAGCCAGTCGGACAAAT | TCTATGGAAGCAGAGCCT | 3 |
| Ind3-12 | GGAATCCCTCCCTTCTTGTC | GGTCGGTAAAGACGGTGAAA | 3 |
| Ind3-15 | GCCATTGATCTTCTGCAGGT | TTTGTTGTCAATGCCCTGTT | 3 |
| Ind3-18 | CGACGCTGTTGATCCTGTTA | GAAATTAAGCAGCGGAAGCA | 3 |
| Ind3-22 | TGCTTACAAGGGTCCAAT | GGAGGTGCCTACCAAGAG | 3 |
| RM6832 | GTTGTAAATGCCTGAGTGC | AAAGAGCTAAACCGCTAGG | 3 |
| Ind3-26 | AGGTCTCGTGTCGTTCATCC | TGGAGGGAGCATGTCTATCA | 3 |
| Ind3-27 | TGCAGTTTGTATTCGGGTTG | cgtggcacatgtggttactt | 3 |
| Ind3-31 | TGGGCTATTATTGGGCTTTG | CGTGGGATAAAACCACCAAG | 3 |
| Ind3-34 | ACACTGGCTACGGCAAAG | TTTGTTCGGGAATAATGATGC | 3 |
| Ind3-36 | GCCATCTTGCCATTTGTTCT | ctctgcttttgctgcctctt | 3 |
| RM5548 | GGTGCAGAGTGATGCAATTC | AACATTAGGGATGAGGCTGG | 3 |
| RM5414 | ACCATGGTTCAAGAGTGAAA | ACAGCTCAACCTGTTGAGTG | 4 |
| RM3471 | AGATCCCGACAGATGGTGAC | AACAGAGGGAGGGAGCAGAG | 4 |
| Ind4-3 | TACACGGTAGACATCCAACA | ATGATTTAACCGTAGATTGG | 4 |
| Ind4-4 | AGTGCTCGGTTTGTTTTC | GTCAGATATAATTGATGGATGTA | 4 |
| Ind4-6 | CTCACAGTTTCTAGGCGGAAA | AGCCGAGTAGGGCTGAATAA | 4 |
| RM3742 | ATCGTCCTCCCGATCTCTCG | CCGCCTCTTCTTATACCCTCTCC | 4 |
| RM1359 | CCAAAGGTCAACGAATTCTA | CGGCTGGTTAATTAATCAAA | 4 |
| RM5424 | TAAAGGTGTCCGACAAGAACACG | GATCGATCTGGAGGATTGAAAGG | 4 |
| RM3367 | GGATCCATCCATCCACTGAC | GGATATGTGCTGCTGTGTGC | 4 |
| Ind4-15 | CGTGGCAATATGGTTCCTTT | TCGGATACGTAAAACGGAAAA | 4 |
| RM1113 | GGGCGCATGTGTATTTCTTC | TGGGGAAAAACCACAAGCC | 4 |
| Ind4-18 | ATGTAACCCGGCCAGAGTG | CCATTAACTGGTCGGAATCG | 4 |
| RM153 | GCCTCGAGCATCATCATCAG | ATCAACCTGCACTTGCCTGG | 5 |
| Ind5-1 | CCTTGATCGATTGCTCTGGT | ACTTTCTCCGTGTTGCTTGC | 5 |
| RM17919 | TATGCTTTCTGTCAGCTTCC | CTGCTGTGAAAGAGTTGACG | 5 |
| RM3777 | TGCTTATGTCGCAAGACTAG | ATCTCACCTCTTCCCTACAA | 5 |
| Ind5-3 | GCTCCCCTCAACTTTTCCTC | TCGGTTGCCTGAATACCTTT | 5 |
| RM1237 | CTCCGCGAGCTTTAGAAGAG | CACATACTCTGGCTCTCCCG | 5 |
| RM8211 | GTTTGGGAAGGAGGAATG | AAGTAGAAACGGCCAACAC | 5 |
| Ind5-11 | CTGCACTGCTGAAATGGAGA | GCTGGGATATCAACCCTACG | 5 |
| RM6054 | CCCTCCGTACGGATACACAC | CTCTTCGGCTTCATCTCCTC | 5 |
| Ind5-14 | GGAGTTCTGGTGGCTTTCC | CAGTTCCCCATTTCCCTCTA | 5 |
| RM3321 | CTGCCTAGCCATAGCCAAAC | CTAGCAGCTTGCGATTGGTG | 5 |
| RM480 | GCTCAAGCATTCTGCAGTTG | GCGCTTCTGCTTATTGGAAG | 5 |
| RM1054 | TGAGTTTCCGGTGTTCCATA | AAGGCAAAGTCGTTCAGCTT | 5 |
| Ind5-16 | GCTCAAGCATTCTGCAGTTG | GCGCTTCTGCTTATTGGAAG | 5 |
| RM508 | GGATAGATCATGTGTGGGGG | ACCCGTGAACCACAAAGAAC | 6 |
| Ind6-1 | GCAATCTAGTAAACTGTTCGAGAAA | TGGAATTTAAACATCCTCAATGC | 6 |
| Ind6-2 | CACCTTTTGTCTAATCAAATCAGTTT | TCTTTGGTGCTGAAAGTATGTAAGA | 6 |
| RM276 | CTCAACGTTGACACCTCGTG | TCCTCCATCGAGCAGTATCA | 6 |
| Ind6-4 | CAGTTAACACCAATCCAATCCA | CCAAATGGGCAGTAGTTTGAA | 6 |
| Ind6-8 | GCGATTATTGAGAGCGAGGA | GCCTCTTGTGGGAAGAACAA | 6 |
| Ind6-10 | TCACCTTTATGGTGCCGAAG | GAAGCTGCTTTTGCTTCCAC | 6 |
| RM3498 | GTGAAAGTCGGTGACGATGG | ACTTAGGGGATCAGGGGATG | 6 |
| Ind6-12 | GGCATTGTAGCCAATCCAGA | AAACACACTCCCCCATGAGA | 6 |
| RM3430 | CATTGCTGCAACTCGATCAC | CGAGAGCCACCTAATCTTGG | 6 |
| RM340 | GGTAAATGGACAATCCTATGGC | GACAAATATAAGGGCAGTGTGC | 6 |
| RM494 | GGGAGGGGATCGAGATAGAC | TTTAACCTTCCTTCCGCTCC | 6 |
| Ind7-1 | TGACTGTTACCCTTACGTGCAG | CGGGATGAAACAGATTCTGAG | 7 |
| RM3325 | GGAGCCCTGAACTTTTTGTG | GGGGAATCCTACTTGCTTCC | 7 |
| RM427 | TCACTAGCTCTGCCCTGACC | TGATGAGAGTTGGTTGCGAG | 7 |
| RM4098 | CGTTTGGATGAAGAAGAAGA | AGTGTTCGTTTCGGATTAGA | 7 |
| RM6574 | AACCTCGAATTCCTTGGGAG | TTCGACTCCAAGGAGTGCTC | 7 |
| Ind7-7 | CCCCATGAGGCCTACACTT | AGCAGCATAATCAGATGAGACG | 7 |
| Ind7-8 | ATCGGTGCCGCTCCTAGAT | CACTCCACAGACATGCAATTT | 7 |
| RM11 | TCTCCTCTTCCCCCGATC | ATAGCGGGCGAGGCTTAG | 7 |
| RM3826 | TTAGCTTTCCTCCAGTCTCC | ACGGGTATCTGAAACACAAC | 7 |
| Ind7-12 | TCCGGCGAGAAAATAAGTGT | TTCGTTAATCCTCACGCAGA | 7 |
| RM234 | ACAGTATCCAAGGCCCTGG | CACGTGAGACAAAGACGGAG | 7 |
| RM1209 | CCCAATGATTTGGTACTGCC | GCCCCAGCTAATTCTTGATG | 7 |
| RM1335 | GCATGCATGAATATGATGG | AGATCGAACAAGAAGAGTGG | 7 |
| RM337 | gtaggaaaggaagggcagag | cgatagatagctagatgtggcc | 8 |
| RM6863 | GCTGCAGAATTAAGGAGAAC | TGCTCAAAATAATCAGCTCC | 8 |
| Ind8-2 | TTCAGAAACGGCATCAATCA | GCATATAAGCCTCAGCATGG | 8 |
| Ind8-4 | CGTGCGGCTTACAAGAGATA | TGAGGCACTAATCATCTTCTCtg | 8 |
| Ind8-8 | TTTTTACCGTGTCGGTACTGC | CTCCAAAACACGGGACCAT | 8 |
| Ind8-10 | CGAAAGAGGAGAGGGGTAGT | CGAAAACGAGAAACAAATA | 8 |
| Ind8-12 | TGCTGCTTATGAGGCTGCTA | GCATCACTGTCCTCAGCATC | 8 |
| Ind8-13 | AACCATGAATGAATCCCTGA | TGCAACTGACATCCTGCAAT | 8 |
| RM3496 | CGCTGAAAATACTGAATTGA | AGATGCATTTATTCCGAAAG | 8 |
| Ind8-14 | TGAATTGAACCTCCGTCCTC | AGAACTGCACCACGAAGCTC | 8 |
| Ind8-15 | CAGCAGAGTCCAGAGAAGAT | GCATAAGATGGCGAGTGA | 8 |
| Ind9-2 | CTTTGGATTCAGGGGGA | AACTTGAAACGGAGGCAG | 9 |
| Ind9-4 | CAATTGGGACAAGTTGAAAACA | GAGAGGACGTCATGGAGGAG | 9 |
| RM444 | gctccacctgcttaagcatc | tgaagaccatgttctgcagg | 9 |
| RM296 | CACATGGCACCAACCTCC | GCCAAGTCATTCACTACTCTGG | 9 |
| Ind9-8 | GCATGTATCGTGGACATGGA | TCCTTGATCAACACCGTCAA | 9 |
| RM566 | ACCCAACTACGATCAGCTCG | CTCCAGGAACACGCTCTTTC | 9 |
| Ind9-9 | GCGAACCGATAAAACTGCTC | AGAGGTGTATCAAAGCAATCGAG | 9 |
| RM242 | GGCCAACGTGTGTATGTCTC | TATATGCCAAGACGGATGGG | 9 |
| Ind10-3 | TGAACAATAAACCACAGAAGCA | CCCTTTATTCCCTCCTTTG | 10 |
| RM311 | TGGTAGTATAGGTACTAAACAT | TCCTATACACATACAAACATAC | 10 |
| Ind10-8 | AATGACAAGGCCGACGATAG | TATTACCCAGGCCAACCTGT | 10 |
| RM8201 | GCCGGCGAGCTACTACTAC | TCTGTTTATAAGCGCAGCAC | 10 |
| Ind10-10 | ATGGAACGCATGACATGAAA | CATCAAGAGGAGGGCAAAAA | 10 |
| Ind10-7 | CCCTAAAAATAGAGCAACCT | ACCCATAATACTACCAATCAAC | 10 |
| Ind10-11 | CTCAGTTGTTGGGGGATGAG | CTTTGGAGATGTGCCAGAGA | 10 |
| Ind10-9 | GTCCCTAGGCCATCTCTTG | GCGAATAGGGGTGGACAG | 10 |
| Ind10-12 | CGACACATGGTGCAGATATGA | TGTGACACGGTGTAGTGACG | 10 |
| RM3451 | CGGCGAGATAACAATTCTCC | GCGTGATGATATGGTATCGG | 10 |
| Ind11-1 | ACAAAGTCTAAGGCCTGaaaaa | GATGTCTTCCGGGTGAGCTA | 11 |
| RM4B | TTGACGAGGTCAGCACTGAC | AGGGTGTATCCGACTCATCG | 11 |
| RM332 | gcgaaggcgaaggtgaag | catgagtgatctcactcaccc | 11 |
| RM1124 | AAGCTATCCCCCTTTTTGGC | AGGGATCGGTAGACCCAATC | 11 |
| Ind11-3 | GTGAATTCATGACGCGAAGA | AGCTTGATGGATGCTCAGGT | 11 |
| RM202 | CAGATTGGAGATGAAGTCCTCC | CCAGCAAGCATGTCAATGTA | 11 |
| Ind11-7 | TGATAAATCCCATACTAAGGATCTG | GCTTAGTCCGAGGCCTTCA | 11 |
| RM5349 | AGGGCATGCTTACATCCAAC | CATTTGCTTCTATGCCCCAG | 11 |
| Ind11-12 | AAGAAAAATATCTATTGAGGAGTG | GGAGGACCATAAATGACGG | 11 |
| Ind12-1 | TCTGGAGAGCTGCAGAAACA | GCAATCTCTGCACTTTGATACC | 12 |
| RM19 | caaaaacagagcagatgac | ctcaagatggacgccaaga | 12 |
| Ind12-2 | TCTCAAGCATGTCAAGGCTTA | ATGAACATGCAGAGCACCAA | 12 |
| Ind12-5 | GAGTTCGGCGACAGTCAGT | TTGAAACATCCACGAATCTCA | 12 |
| RM1337 | GTGCAATGCTGAGGAGTATC | CTGAGAATCTGGAGTGCTTG | 12 |
| Ind12-12 | CGGTCGTACGTGTAGCAAGA | TGATGGCCAAGAATTTAGGG | 12 |
| Ind12-14 | CCGCCGAGAAGAAACAAAG | CCCAAGAACAGGATTACA | 12 |
| RM17 | TGCCCTGTTATTTTCTTCTCTC | GGTGATCCTTTCCCATTTCA | 12 |
| Ind12-15 | TGTGCTTCTGGTGAACAACTTT | CCAAATGGCGGGTAATCATA | 12 |
